# Emergent Laparoscopic Repair of a Spigelian Hernia: Case Report and Review of the Literature

**DOI:** 10.1155/2013/197561

**Published:** 2013-04-10

**Authors:** Reid Barker, Richdeep S. Gill, Avneet S. Brar, Daniel W. Birch, Shahzeer Karmali

**Affiliations:** ^1^Faculty of Medicine & Dentistry, University of Alberta, Edmonton, AB, Canada T5H 3V9; ^2^Department of Surgery, University of Alberta, Edmonton, AB, Canada T5H 3V9; ^3^Department of Family Medicine, University of Calgary, Calgary, AB, Canada T2N 1N4; ^4^Center for the Advancement of Minimally Invasive Surgery (CAMIS), Royal Alexandria Hospital, Edmonton, AB, Canada T5H 3V9

## Abstract

A spigelian hernia is a protrusion through an anterior abdominal wall defect along the linea semilunaris. The traditional method of repair consists of an open surgical technique requiring a lengthy abdominal incision to allow visualization of the defect. However, with the emergence and availability of laparoscopic techniques, a minimally invasive approach is feasible. Only eight prior case reports have documented emergent laparoscopic repair of a spigelian hernia. We describe the first successful laparoscopic repair of a spigelian hernia in an emergent setting at our institution.

## 1. Introduction

A spigelian hernia is the result of an anterior abdominal wall defect. A semilunar line along the rectus sheath was first described by Adrian van der Spieghel. The first hernia arising from this line was described by anatomist Josef Klinkosh. A spigelian hernia is an anterior wall defect in the transversus abdominis muscle close to the arcuate line of Douglas. It occurs laterally to the lateral border of the anterior rectus sheath along the semilunar line, where a transition from muscle to aponeurosis occurs [1]. Spigelian hernias represent about 1-2 percent of all abdominal wall hernias [2].

The traditional method of repair has been through an open incision, but, with the growth and availability of laparoscopic equipment, the laparoscopic technique is starting to play a greater role. Emergent cases in which the repair has been completed laparoscopically are now being described in the literature [1, 3]. Over the past 20 years laparoscopic surgeries have become increasingly utilized with major advantages, including shorter length of hospital stay and lower postoperative complications such as infections, hernias, and scarring. Spigelian hernias offer a unique challenge to general surgeons: they require a surgeon well-versed with laparoscopic techniques, specifically hernia repairs but are fairly rare so the surgeon may not have encountered this problem specifically in an emergent setting. This case report will present a laparoscopic repair performed in an emergent situation with a postoperative complication.

## 2. Case Report

A 62-year-old female presented to the emergency room with obstructive gastrointestinal symptoms including nausea and vomiting. Three weeks prior to presentation, the patient had undergone a laparoscopic cholecystectomy and developed a postoperative gastric volvulus. This was decompressed three days after cholecystectomy with a gastrostomy tube. The gastrostomy tube was connected to suction, relieving some of the patient's abdominal symptoms.

On presentation the patient was tachycardic, with a heart rate of 123 beats/minute and a blood pressure of 107/73. She was afebrile. Her abdominal exam was significant for both right upper and lower quadrants pain, but she did not exhibit any abdominal guarding or rebound tenderness. 

The patient's physical exam indicated the presence of an incarcerated hernia, but it could not be determined at the bedside whether this was a spigelian or ventral hernia. A computed tomography (CT) scan was ordered for a definitive diagnosis, as well as to determine the location and contents of the lesion while the patient was stable.

**Figure 1:**
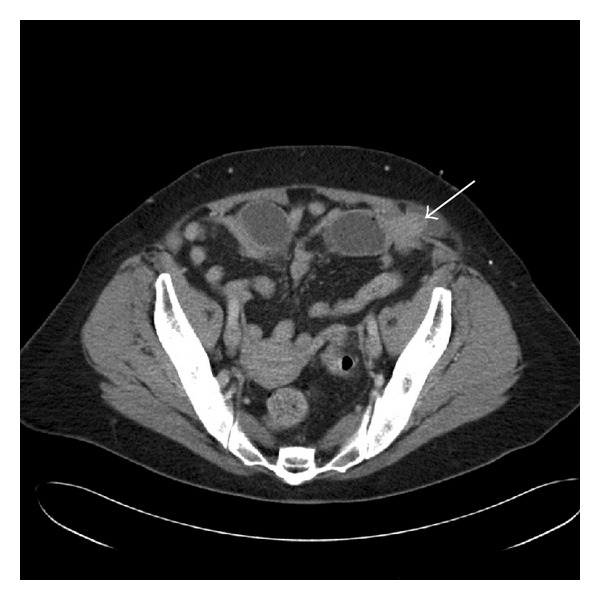
Computed tomography (CT) of the abdomen demonstrating a left-sided spigelian hernia with incarcerated loop of small bowel (white arrow points to loop of small bowel incarcerated).

The patient was initially treated with intravenous fluids and broad-spectrum antibiotics prior to CT scanning. The CT scan demonstrated a high-grade small bowel obstruction caused by an entrapped loop of small bowel within a left-sided spigelian hernia (see Figure 1). Informed consent was obtained, and the patient was taken to the operating room for emergency laparoscopic repair of her spigelian hernia. The laparoscopic approach involved placement of the patient in the supine position with both arms tucked. Pneumoperitoneum was established using a veress needle in the midline at the umbilicus. Three ports were inserted under direct vision (a 5 mm port in the midline, a 10 mm port in the right midclavicular line, and a 5 mm port in the right suprapubic region). A spigelian hernia was noted on the left lower abdominal wall and measured about 3.5 cm in its greatest diameter. Fortunately, the uncompromised loop of small bowel was reduced back into the peritoneal cavity using a hunter atraumatic laparoscopic grasper. Absorbable mesh was than introduced into the peritoneal cavity and the defect repaired. A spinal needle was repeatedly introduced around the hernia site, mapping out the size of the defect and the area on the abdominal wall to determine the mesh size needed. The absorbable mesh was cut to provide 3 cm of overlap around the entire hernia defect and then attached using both tacks and sutures. 

This patient was discharged on her third postoperative day but returned to hospital one month later. She had pain at her second surgical site and was noted to have a fluid collection on CT scan caused by a postoperative seroma. The patient was treated conservatively with intravenous antibiotics, and followup ultrasound demonstrated reduction in size of the fluid collection. The patient's pain also improved during the short course of antibiotic treatment. The patient was seen in followup at 3 months and 6 months postoperatively, and no sign of clinical recurrence was identified.

## 3. Discussion

Historically, preoperative diagnosis of spigelian hernias has been difficult based purely on history and physical exam findings. Symptoms are often nonspecific and are likely to be pain and nausea. Vomiting and altered bowel habits may also be of the presenting complaints [11]. Physical exam is most helpful when a palpable lump is present, although the swelling caused by the underlying hernia is often masked by the overlaying external oblique aponeurosis [1]. While this lump can sometimes be made more prominent by having the patient sit up and forward flex at the lumbar spine, a patient's body habitus may increase the difficulty of determining diagnosis due to physical exam alone. A retrospective study of 76 patients at the Mayo clinic [12] demonstrated the difficulty of diagnosing these hernias purely by means of physical findings. A definite and identifiable abnormality could be found in only 64% of spigelian hernia patients who presented with abdominal symptoms. The other 36% could not be diagnosed based on careful physical examination alone. An examining physician should therefore maintain a high index of suspicion for these abdominal defects, as imaging may be required to completely exclude it from a differential diagnosis.

Risk factors are similar to other abdominal wall hernias and include COPD, obesity, collagen disorders, and other abdominal wall defects [11]. Some authors have suggested that almost 50% of patients with spigelian hernias have had previous abdominal operations, either open or laparoscopic. Other risk factors include sex (male : female—1 : 1.18) [13], with most diagnoses being made between the fourth and seventh decades of life [14].

The differential diagnosis is extensive and includes appendicitis, an abdominal wall or intra-abdominal abscess, abdominal wall hematoma, diverticulitis, and neoplasms arising from either the abdominal wall or intra-abdominal organs [15]. With the advent of cross-sectional imaging and ultrasound, a preoperative diagnosis has become more common [16]. The role of MRI is still being defined to help clinicians with this difficult diagnosis. However, once discovered, spigelian hernias most often necessitate surgical repair due to their usual presentation as a narrow and inflexible opening that leads them to be prone to incarceration and strangulation [17].

We were only able to retrieve eight case reports that have discussed the laparoscopic repair of a spigelian hernia in an emergent setting [1, 4–10, 18]. Surgical techniques are variable throughout the eight cases, but, in each case, the surgeons decided to close the defect with mesh attached by staples, sutures and/or tacks. These eight case reports also had variable followup ranging from none reported in two cases [8, 9] to a three-year followup after hospital discharge in one case [6, 18]. 

A potential advantage of laparoscopic repair is to reduce the length of hospital stay. From the eight cases that reported time to discharge, the mean time was 2.75 days (Table 1). Our patient was discharged on her third postoperative day but did require readmission one month later for treatment with intravenous antibiotics for suspected infected seroma. This represents the first reported complication following laparoscopic repair of a spigelian hernia in an emergent situation. All previously described cases used mesh to repair the hernia defect, with surgeons using staples, sutures, or tacks or a combination of these. Our case used tacks initially to attach the mesh to the peritoneal cavity and then sutures it to ensure its stability.

Due to the rarity and elusive diagnosis of the spigelian hernia, the paucity in the literature is understandable. A randomized prospective trial comparing open and laparoscopic surgical repairs of spigelian hernias reported a marked decrease in morbidity (*P* < 0.05) and hospital stay (*P* < 0.001) with laparoscopic repair [19]. The study compared the outcomes of 22 patients (11 laparoscopic and 11 open techniques) who were randomly assigned to either open or laparoscopic hernia repair. The study was conducted exclusively on elective patients and excluded emergent cases. However, it demonstrates the efficacy and safety of the laparoscopic approach, a belief mirrored by Skouras et al. in their comprehensive review of laparoscopic spigelian hernia repair [17].

It is difficult to know whether the complication seen in our case is a common occurrence in this procedure since the number of cases described in the literature is limited. However, the authors do not believe that this indicated a higher risk of treating these emergent cases laparoscopically, as laparoscopic procedures within the emergent setting carry a lower risk of wound complications when compared to open surgeries in other procedures such as appendectomies [20]. 

A recent review of the literature concluded that laparoscopic spigelian hernia repair was safe and feasible provided a well-trained laparoscopic surgeon is available to perform the operation [1]. However, the authors of this review did comment on the need to continue to publish similar case reports given the small sample size of laparoscopic spigelian hernia repairs done in an emergent setting. Specifically, cases in which complications are noted must be reported to help better determine whether any surgical type, technique, or materials offers an advantage for better patient outcomes.

## Figures and Tables

**Table 1:** Summary of the literature review of patients undergoing laparoscopic repair in an emergent setting.

Study	No. of pts	Followup (months)	Complications	Mesh fixation method	Time to discharge (days)
Leff et al. [1]	1	4	0	Staples/sutures	2
Amendolara [4]	1	3	0	—	1
Habib and Elhadad [5]	1	12	0	Staples	2
Saber et al. [6]	2	36	0	Sutures/tacks	5, 3
Lopez-Tomassetti Fernandez et al. [7]	1	10 months	0	Tacks	3
Novell et al. [8]	1	—	0	Staples	—
Yau et al. [9]	1	—	—	Staples	4
Subramanya et al. [10]	1	6	0	Tacks	2

—: not reported.
